# *miR-146a* Expression Level as a Novel Putative Prognostic Marker for Acute Promyelocytic Leukemia

**DOI:** 10.1155/2014/150604

**Published:** 2014-08-05

**Authors:** Lan Xu, Hua Zhong, Haixia Wan, Fang-yuan Chen, Jihua Zhong, Fei Xiao, Jia Liu, Lijing Shen

**Affiliations:** Department of Hematology, Ren Ji Hospital, School of Medicine, Shanghai Jiao Tong University, Shanghai 200127, China

## Abstract

*Background*. Although the curative rate for acute promyelocytic leukemia (APL) has been improved over decades, long-term prognosis is still poor. The genetic pathways that regulated cell lineage fate during the development of APL remain unclear.* Methods*. We investigated the correlations of* miR-146a* expression with its target gene Smad4 and the biological behaviors of NB_4_ cells. We also analyzed their expression in clinical samples from APL patients.* Results*. *miR-146a* influenced apoptosis and proliferation in NB_4_ cells. *miR-146a* influenced endogenous Smad4 protein levels in APL cells. *miR-146a* expression levels were positively correlated with white cell counts and PML/RAR*α* fusion protein expression. *miR-146a* expression levels were negatively correlated with Smad4 protein and the helper T cell (Th)/the suppressor T cell (Ts) ratio in these patients. *Conclusions*. These findings indicated that *miR-146a* played an important role in the development of APL in part through the repression on Smad4 protein expression. *miR-146a* functioned as an oncogene and may be a novel prognostic biomarker in APL.

## 1. Introduction

Acute promyelocytic leukemia (APL) has been identified as an M_3_ subtype of acute myelogenous leukemia (AML) by the French-American-British (FAB) classification. APL is characterized by maturation arrest at the promyelocytic stage [1]. A specific t (15; 17) chromosomal translocation encodes a promyelocytic leukemia (PML) and retinoic acid receptor-*α* (RAR*α*) fusion protein to form PML/RAR*α*, an oncogenic protein found in approximately 10%–25% of adults with AML [2]. PML/RAR*α* interferes with the process of myeloid differentiation by repressing the transcription of retinoid acid- (RA-) responsive genes.

Although the outcomes of APL have been dramatically improved since the successful introduction of all-trans retinoic acid (ATRA), arsenic acid, and combined anthracycline-based chemotherapy, more than 10% of newly diagnosed APL patients die of the disease; moreover, the 5-year cumulative incidence of relapse is around 15% in high-risk subgroups [3]. Furthermore, the specific genes and pathways that regulate lineage fate during APL development remain unclear.

MicroRNAs (miRNAs) are a group of highly conserved non-protein-coding RNAs comprised of about 19–25 nucleotides. miRNAs can regulate the expression of a variety of genes by binding the 3′ untranslated regions (3′ UTRs) of messenger RNAs (mRNAs) in a sequence-specific manner to regulate mRNA translation or degradation in eukaryotic cells [4], ultimately affecting cell proliferation, apoptosis, development, and differentiation [5].

*miR-146a* was first identified as having a role in the innate immune and inflammatory response to microbial infection [6]. Later studies showed that* miR-146a* is expressed at relatively high levels in bone marrow (BM) CD34^+^ progenitors from healthy donors but is found at low levels in AML patients and even lower levels in monocytes, granulocytes, erythrocytes, and megakaryocytes from the peripheral blood or BM of healthy donors [7]. *miR-146a* is strongly upregulated during megakaryopoiesis in mice [8], and dysregulation of* miR-146a* has been found in APL cells following retinoic acid (RA) induction [9–12]. Our previous study confirmed the reduced expression of* miR-146a* in NB_4_ cells following treatment with ATRA [13]. However, the role of* miR-146a* in the clinical progression of APL remains unknown.

In this study, we investigated the correlations of* miR-146a* expression with the expression of its target gene Smad4 and the biological behaviors of NB_4_ cells, such as proliferation and apoptosis. To further elucidate the role of* miR-146a* in APL, we analyzed its expression in samples from 32 APL patients for whom clinical data were available. Interestingly, the expression levels of* miR-146a* were positively correlated with white blood cell (WBC) counts in the peripheral blood and expression of the PML/RAR*α* fusion protein. *miR-146a* expression levels were also negatively correlated with Smad4 protein and the helper T cell (Th)/the suppressor T cell (Ts) ratio in these patients. These results indicated that* miR-146a* functioned as an oncogene in APL and may be a potential biomarker for malignancy.

## 2. Materials and Methods

### 2.1. Cell Culture and Transient Transfection with* miR-146a* Mimics

The human promyelocytic cell line NB_4_ was a gift from Shanghai Institution of Hematology and was cultured in RMPI 1640 medium with 10% fetal calf serum (FCS; Gibco, BRL, UK) at 37°C in a 5% CO_2_ humidified incubator. On the day of transfection, 5 × 10^6^ cells/mL were plated in 6-well plates with RMPI 1640 supplemented with 10% FBS. Transfections were carried out with 100 nM DMEM-diluted pre-miR* miR-146a* precursor, pre-miR negative control, anti-pre-miR* miR-146a* precursor, or anti-pre-miR negative control (Ambion, Carlsbad, CA, USA) using siPORT NeoFX Transfection Agent (Ambion, Carlsbad, CA, USA). Twenty-four hours after transfection, the medium was replaced, and the cells were cultured for another 24 h. To monitor the transfection efficiency of the miRNAs, pre-miR* has-miR-1* precursor and anti-miR* has-let-7c* miRNA inhibitor were transfected into NB_4_ cells in parallel experiments according to the manufacturer's instructions.

### 2.2. Cell Proliferation, Apoptosis, and Cell Cycle Analyses

Live cell proliferation assays were carried out by trypan blue dye exclusion using a Bio-Rad automatic cell counter (Bio-Rad, Berkeley, CA, USA). The number of NB_4_ cells was calculated in triplicate after 24 or 48 h of culture.

Apoptotic cells were detected with an Alexa Fluor 488 annexin V/Dead cell apoptosis kit (Invitrogen, Carlsbad, CA, USA) by flow cytometry (BD Biosciences, Franklin Lakes, NJ, USA). Early apoptotic cells were defined as Annexin-V-positive/propidium iodide- (PI-) negative cells. The experiments were repeated 3 times.

For analysis of the cell cycle distribution, NB_4_ cells were washed 3 times with cold phosphate-buffered saline (PBS), fixed with 70% ethanol, and incubated at −20°C for more than 12 h. Before examination, the cells were washed with cold PBS and stained with 0.5 mL PI staining buffer (200 mg/mL RNase A and 50 *μ*g/mL PI in PBS). The mixture was incubated at 37°C for 30 min in the dark, and cell cycle distribution was analyzed by flow cytometry (BD Biosciences). The experiments were repeated 3 times.

### 2.3. miRNA and mRNA Extraction and Real-Time Quantitative Polymerase Chain Reaction (RTq-PCR)

miRNA was extracted with a mirVana miRNA isolation kit (AM1560, Applied Biosystems, Foster City, CA, USA). Mature* miR-146a* was detected by TaqMan miRNA assay as previously reported [13], using a TaqMan_MicroRNA Reverse Transcription Kit (4366597, Applied Biosystems) and PCR 9700 sequence detection system (Applied Biosystems). U48 was used as an internal control. miRNA levels were expressed as relative 2^−ΔCT^ values.

mRNAs of other genes (*Smad4*,* PML/RAR* *α*,* ABL*, and* GAPDH*), excluding miRNAs, were extracted using a QIAampRNA blood mini kit (Qiagen, Valencia, CA, USA), and 10 *μ*g mRNA was used in each reverse transcription reaction, carried out with Superscript II reverse transcriptase and random primers (Invitrogen). Gene expression was determined by RTq-PCR as previously described [14] on a PCR 9700 sequence detection system.* ABL* and* GAPDH* were used as internal controls. mRNA levels were expressed as relative 2^−ΔCT^ values.

### 2.4. Western Blot Analysis

A total of 5 × 10^6^ cells of each experimental group were harvested and subsequently were washed twice with phosphate-buffered saline (PBS). Cells were then lysed with lysis buffer (300 mM NaCl, 0.5% NP-40, 1 mM DTT, 200 mM PMSF, protease inhibitor tablet) for 20 minutes. The protein concentration of the cell lysates was quantified using a bicinchoninic acid (BCA) protein Assay Kit (Kangchen, China). 20 *μ*g protein extracted from each experimental sample was separated by sodium dodecyl sulfate-polyacrylamide gel electrophoresis (SDS-PAGE), transferred to polyvinylidene difluoride (PVDF) membranes and blotted with rabbit polyclonal anti-Smad4 (1 : 5000, Cell Signaling Technology, Danvers, MA, USA) or rabbit monoclonal anti-GAPDH (1 : 2000, Cell Signaling Technology) antibodies. Protein bands were visualized with the use of enhanced chemiluminescence reagent (Pierce), according to the manufacturer's instructions, and band intensities were analyzed using Bandscan 5.0 (Glyko, Hayward, CA, USA).

### 2.5. Patients, Sample Collection, and Therapeutic Methods

Thirty-two APL patients and ten iron deficiency anemia patients as control who attended Renji Hospital were enrolled in this study. The clinical characteristics of these patients are shown in Table 1. Bone marrow was collected from patients at diagnosis or after therapy. APL patients were treated with ATRA (30 mg/m^2^) and daunorubicin (DNR; 60 mg/m^2^)/idarubicin (IDA; 8 mg/m^2^) daily for 3 days. Arsenic trioxide was used at 10 mg daily until patients achieved complete remission. The details of these patients including treatment protocol can be found in Table 2. Protein extracts from 14 patients were used for western blotting, including 6 pairs of samples taken before and after therapy. Written informed consent for participation in this study was obtained from all patients. The study was approved by the Ethics Committee of Renji Hospital (number 81270626).

### 2.6. Statistical Analyses

To investigate whether* miR-146a* expression correlated with clinical quantitative variables (e.g., WBC counts), we used Pearson correlation analysis within SPSS 16.0 (SPSS Inc., Chicago, IL, USA). The Mann-Whitney *U* test was carried out to assess the differential expression of* miR-146a* for statistical significance. Data from western blotting were analyzed by paired *t*-tests using SAS version 12.6. All results were presented as the mean ± standard deviation (SD). *P* values of less than 0.05 were considered statistically significant.

## 3. Results

### 3.1. Ectopic Expression of* miR-146a* Affected Cell Apoptosis and Proliferation but Did Not Affect the Cell Cycle in NB_4_ Cells Lines

**Figure 1:**
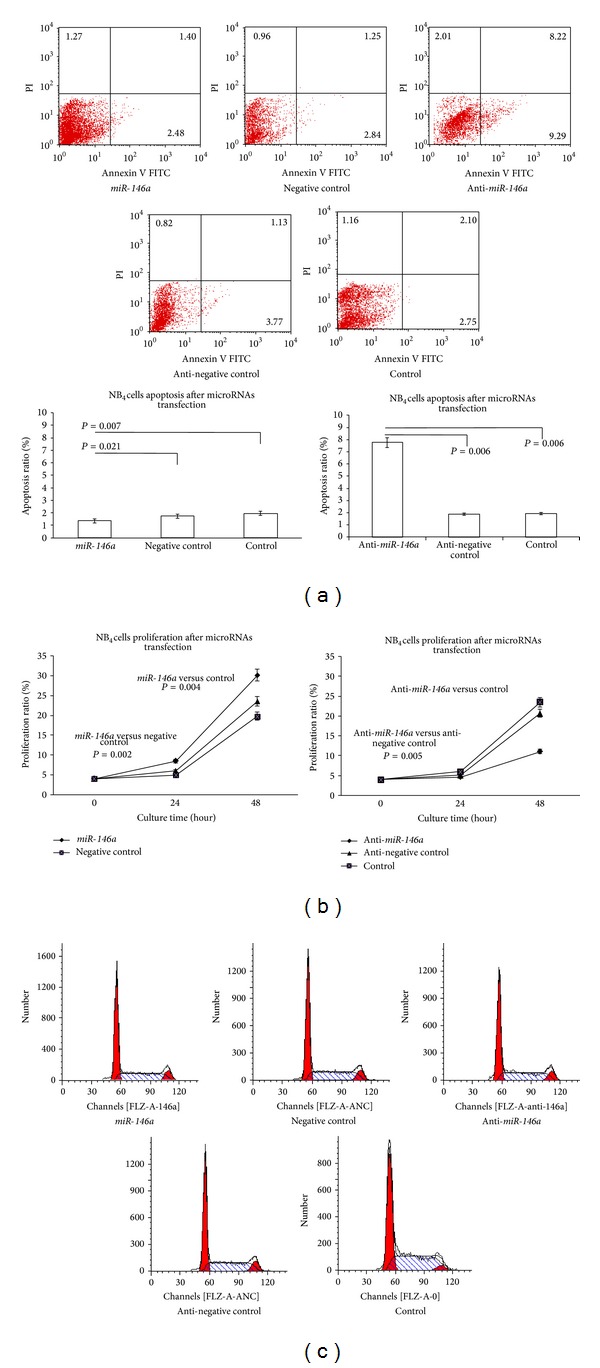
The functional role of* miR-146a*. (a) Forced expression of* miR-146a* by transfection of cells with pre-*miR-146a* with lipidosome significantly inhibited apoptosis, whereas downregulation of* miR-146a* by anti-pre-miR* miR-146a* inhibitor significantly increased apoptosis in NB_4_ cells. (b) Forced expression of* miR-146a* significantly increased, whereas downregulation of* miR-146a* significantly decreased cell proliferation in NB_4_ cells. (c) The cell cycle was not affected after ectopic expression of* miR-146a*. Normalized mean values of 3 independent experiments and standard errors (means ± SDs) are shown. *, *P* ≤ 0.05; **, *P* ≤ 0.001 (paired *t* test).

To examine the functional role of* miR-146a* in APL cells, we transfected* miR-146a* into NB_4_ cells and collected the cells 48 h after transfection. The results showed that forced expression of* miR-146a* significantly inhibited apoptosis and increased cell viability in NB_4_ cells, while knockdown of* miR-146a* expression using an anti-*miR-146a* inhibitor increased apoptosis and reduced cell viability significantly (Figures 1(a) and 1(b)). Overexpression of* miR-146a* in NB_4_ cells did not affect the cell cycle distribution (Figure 1(c)). The expression of* miR-146a* before and after transfection was confirmed by RTq-PCR (data not shown). Thus,* miR-146a* may function as an oncogene in leukemogenesis.

### 3.2. Exogenous Expression of* miR-146a* Affected Endogenous Smad4 Protein Expression in APL Cells

**Figure 2:**
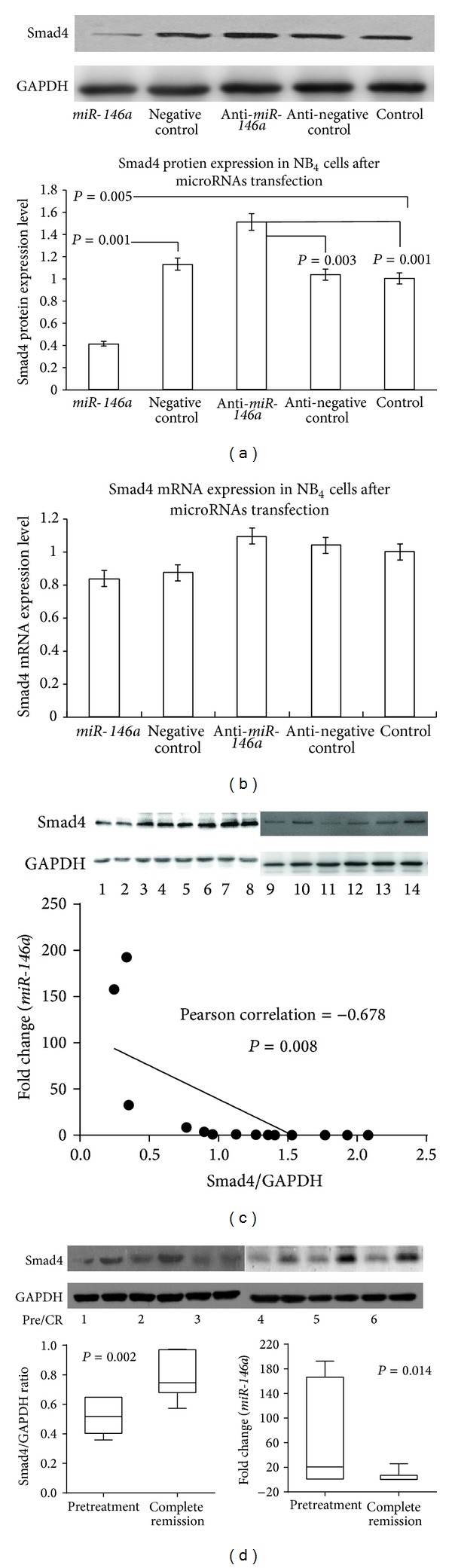
Exogenous* miR-146a* and endogenous Smad4 expression. (a) Western blot analysis of Smad4 protein expression in NB_4_ cells after transfection with* miR-146a* duplex,* miR-146a* inhibitor duplex, or scrambled duplex. (b) RTq-PCR analysis of* Smad4* gene expression in NB_4_ cells after transfection with* miR-146a* duplex,* miR-146a* inhibitor duplex, or scrambled duplex. (c)* miR-146a* was inversely correlated with Smad4 protein expression in samples from 14 APL patients. (d)* miR-146a* and Smad4 protein expression analysis in samples from 6 APL patients at diagnosis and after complete remission using RTq-PCR and western blotting. Normalized mean values of 3 independent experiments and standard errors (means ± SDs) are shown. *, *P* ≤ 0.05; **, *P* ≤ 0.001 (paired *t* test).

As shown in our previous research, Smad4 protein expression was repressed by more than 30% in* miR-146a*-transfected 293T cells [13]. We overexpressed* miR-146a* to confirm the effects of* miR-146a* on Smad4 protein expression in NB_4_ cells. The results showed that* miR-146a* significantly reduced Smad4 protein levels but did not affect* smad4* mRNA expression, as compared with the scramble control group. In contrast, transfection with* miR-146a* inhibitor increased Smad4 protein (Figures 2(a) and 2(b)), indicating that* miR-146a* regulated the expression of Smad4 protein in NB_4_ cells. To confirm the relation in primary leukemia cells, the expression levels of* miR-146a* and Smad4 were examined in 14 APL samples. The results showed that Smad4 expression was inversely correlated with* miR-146a* expression in APL samples (Figure 2(c)).

As we previously demonstrated in NB_4_ cells, ATRA could suppress* miR-146a* expression, which subsequently increased the expression of Smad4 protein [13]. To study the effects of ATRA on* miR-146a* and Smad4 expression levels in primary cells, 6 pairs of samples from APL patients before and after ATRA treatment were analyzed. The results showed that* miR-146a* expression decreased, while Smad4 protein levels increased after treatment with ATRA, as compared to matched samples before therapy (Figure 2(d)).

These data demonstrated that the negative correlation between* miR-146a* and Smad4 protein level may be an indicator of disease progression and treatment outcomes.

### 3.3. *miR-146a* Expression Was Associated with the Clinical Characteristics of Patients

**Figure 3:**
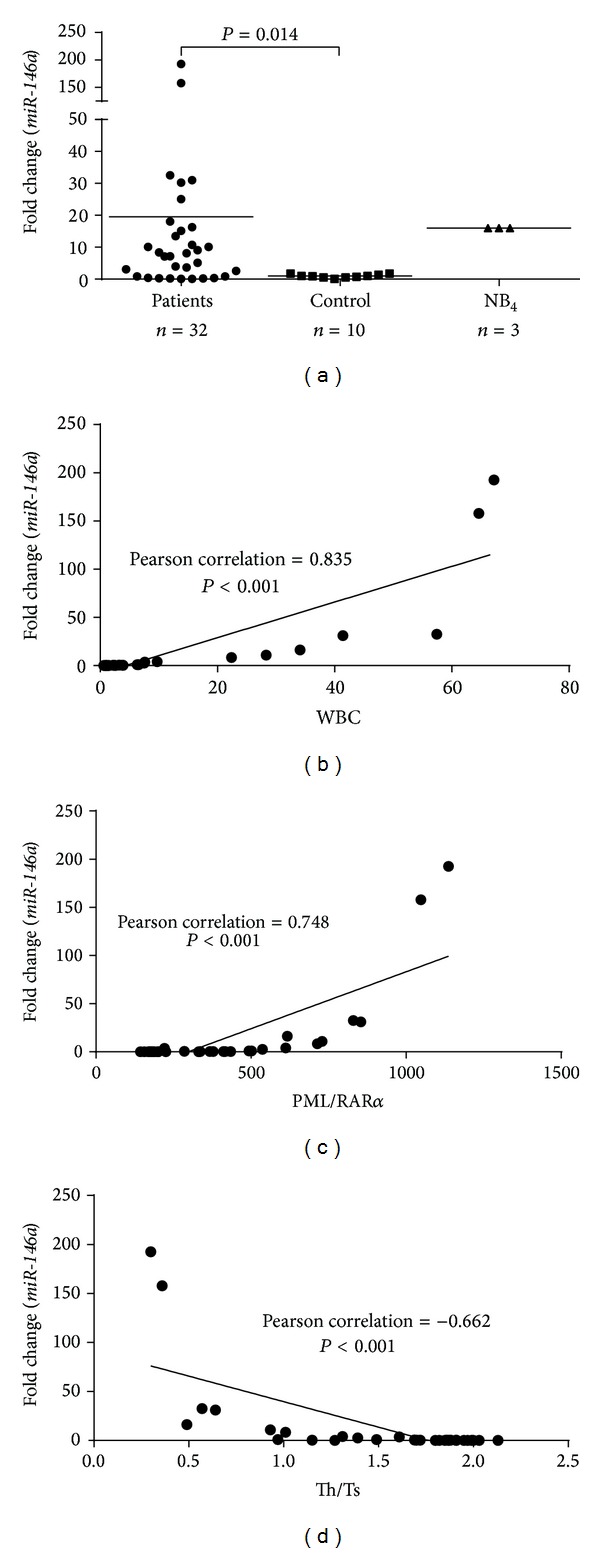
*miR-146a* expression and clinical characteristics of 32 patients. (a)* miR-146a* levels in APL patients, controls, and NB_4_ cells. (b)* miR-146a* levels were positively correlated with white blood cell (WBC) counts. (c)* miR-146a* was positively correlated with PML/RAR*α* gene expression. (d)* miR-146a* expression was negatively correlated with the Th/Ts ratio.

Next, we investigated the correlations between* miR-146a* expression and patient characteristics, including age, WBC count, blast cell percentage in the bone marrow or peripheral blood, PML/RAR*α* expression levels, and Th/Ts ratios. Compared to the controls, we found average* miR-146a* expression levels were higher in APL patients (Figure 3(a)). We found a positive correlation between* miR-146a* expression and peripheral WBC counts (Figure 3(b)). However, no correlations were found between* miR-146a* expression and age or blast percentage in peripheral or bone marrow (data not shown).

To investigate the correlations between* miR-146a* and PML/RAR*α* and Th/Ts, 32 samples from patients with APL were examined. The results showed that patients with higher* miR-146a* expression exhibited higher levels of PML/RAR*α* (Figure 3(c)), but lower Th/Ts ratios (Figure 3(d)).

## 4. Discussion

Recent studies have mainly focused on analyzing the function of* miR-146a* in hematopoietic cell differentiation; these data have indicated that* miR-146a* expression is finely tuned during cell differentiation and* miR-146a-*mediated expression of target genes plays an important role in this process [15]. Moreover, one study demonstrated that overexpression of* miR-146a* in mouse hematopoietic stem/progenitor cells results in a transient myeloid expansion in vivo [16].

*miR-146a* and its predicted target gene Smad4 were identified in a previous study using luciferase assays [13]. In our previous study,* miR-146a* expression was decreased during retinoid acid induction in NB_4_ cells, accompanied by upregulation of Smad4 protein. These results suggested that* miR-146a* may play specific roles in APL genesis or ATRA-induced cell differentiation. Elucidating the role of* miR-146a* will improve our understanding of the malignant progression of APL.

In our present study, transfection with* miR-146a* mimics increased proliferation, while inhibition of* miR-146a* expression resulted in decreased cell growth and increased apoptosis in NB_4_ cells. However, ectopic expression of* miR-146a* had no effect on cell cycle in NB_4_ cells. These findings are consistent with several previous studies. Starczynowski et al. investigated the role of* miR-146a* in hematopoiesis by using retroviral infection and overexpression of* miR-146a* in mouse hematopoietic stem/progenitor cells, followed by bone marrow transplantations. The transplantation of these cells resulted in a transient myeloid expansion [16]. Moreover,* miR-146a* has been found to play an antiapoptotic role during T cell activation [17].

Some other results were inconsistent with our results. Overexpression* miR-146a* in hepatic stellate cell (HSC) inhibited cell proliferation by enhancing cell apoptosis [18]. Treating HL60 cells with demethylating agents increased* miR-146a* expression, and forced expression of* miR-146a* decreases cell proliferation in HL60 cells [19]. Chen et al. also showed that* miR-146a* was upregulated during phorbol 12-myristate 13-acetate (TPA)-induced differentiation of HL-60 cells [20].* miR-146a* has also been shown to be important in the development and maintenance of cancers, including anaplastic thyroid cancer, pancreatic carcinoma, gastric cancer, breast cancer, prostate cancer, glioma, and cervical cancer [21–28]. All of these data show* miR-146a* expression, function, and regulation in cancers or hematopoietic malignancies may vary in terms of the types of tissues or cells affected. Additionally,* miR-146a* expression in different cancers or tissues is thought to be regulated primarily by point mutations, histone deacetylation, or promoter methylation [29].

The transforming growth factor (TGF)-*β* signaling pathway and its downstream target proteins, including Smads, are known to repress cell proliferation in leukemia cells. Inactivation of the TGF-*β*/Smad signaling pathway can induce the development of leukemia in humans. Smad4 is the only Co-Smad that has been identified in mammals and is known to play a key role in the TGF-*β*1/Smad signaling cascade [30]. Furthermore, mutations in Smad4 can block nuclear translocation of this gene, which is associated with the pathogenesis of acute myelogenous leukemia [31]. Our data showed that transient transfection with* miR-146a* mimics suppressed Smad4 protein expression in NB_4_ cells, while transfection with an* miR-146a* inhibitor increased Smad4 protein expression in these cells. Like other miRNAs,* miR-146a* can modulate cellular activities, such as cell proliferation, differentiation, and migration, by targeting mRNAs encoding specific proteins. Overexpression of* miR-146a* in a model of osteoarthritis (OA) was accompanied by downregulation of Smad4 in vivo [32]. In this OA model study,* miR-146a* was proposed to contribute to OA pathogenesis by impairing the TGF-*β* signaling pathway through targeted inhibition of Smad4 in cartilage [32]. Interestingly,* miR-146a* overexpression in HSC can also reduce Smad4 protein levels without affecting its mRNA expression [18]. These discoveries have provided insights contributing to our understanding of the mechanisms involved in* miR-146a*/Smad4 signaling transduction.

*miR-146a* expression is significantly downregulated during monocytic differentiation in NB_4_ cells in response to chemical inducers vitamin D and phorbol 12-myristate 13-acetate (PMA) [33]. Our previous study showed that* miR-146a* is downregulated during NB_4_ differentiation induced by ATRA [13]. In the current study,* miR-146a* expression was decreased, accompanied by increased Smad4 protein expression in APL patients exhibiting complete remission. These data suggested that* miR-146a* expression was correlated with APL cell differentiation.

In our analysis,* miR-146a* was found to be positively correlated with WBC counts in patients, which is consistent with our in vitro data. In another study,* miR-146a* was overexpressed in hematopoietic stem cells, and the modified cells were then transplanted into recipients' bone marrow. Transplantation of these cells resulted in a transient myeloid expansion [16]. This result also showed that* miR-146a* indeed affected cell proliferation. PML/RAR*α* complexes can bind to the predicted PML/RAR*α* binding site in the promoter region of* miR-146a*, and the repressive effects of PML/RAR*α* complexes can be blocked by ATRA, even in primary blast cells [11]. In our study, expression of the PML/RAR*α* fusion gene was decreased after patients achieved complete remission following retinoid acid induction, and* miR-146a* expression decreased simultaneously, indicating that* miR-146a* was regulated by PML/RAR*α* in the leukemogenesis of APL.

*miR-146a* is abundantly expressed in regulatory T cells and T helper type-1 cells as compared with naïve mature T cells and T helper type-2 [34].* miR-146a* contributes to the regulation of the type I interferon signal transduction pathway, which is important for immune surveillance [17, 21]. Our data showed a negative correlation between* miR-146a* and the Th/Ts ratio in APL patients (Figure 3(c)), suggesting that overexpression of* miR-146a* may impair immune surveillance in APL patients.

In conclusion, our data demonstrated that* miR-146a* played an important role in APL cells by increasing cell proliferation and inhibiting cell apoptosis, at least in part via the tumor-suppressor Smad4.* miR-146a* expression was associated with peripheral WBC counts, Th/Ts ratios, and PML/RAR*α* expression levels in APL patients, suggesting that* miR-146a* may represent a novel biomarker for APL and could be used to evaluate the effectiveness of chemotherapy for APL.

## Figures and Tables

**Table 1:** Clinical characteristics of APL patients.

Characteristic	Mean ± SD
Age	42.22 ± 2.82
Gender (male/female)	13/19
WBC count (10^9^/L)	12.22 ± 3.45
BM blast cell percentage (%)	84.70 ± 1.89
PML/RAR*α*	419.81 ± 48.26
Th/Ts ratio	1.48 ± 0.10

**Table 2:** Clinical variables for controls and APL patients.

Patients ID	Gender	Age	WBC (×10^9^/L)	PML/RARa : ABL relative ratio	Th/Ts	Smad4/GAPDH	Therapy
Control							
C1	M	44	6.32	NA	0.98	NA	NA
C2	F	34	4.67	NA	1.03	NA	NA
C3	F	59	5.96	NA	0.61	NA	NA
C4	F	28	11.30	NA	1.31	NA	NA
C5	M	24	8.35	NA	1.87	NA	NA
C6	F	63	5.90	NA	1.55	NA	NA
C7	F	30	7.40	NA	1.07	NA	NA
C8	M	38	3.76	NA	1.12	NA	NA
C9	F	68	7.75	NA	2.05	NA	NA
C10	M	35	4.81	NA	2.11	NA	NA
APL							
A1	F	53	0.80	174	2.03	1.4061	ATRA + ATO + DNR
A2	F	56	3.20	285	1.69	1.2696	ATRA + ATO + IDA
A3	F	67	22.40	713.5	1.01	0.7683	ATRA + ATO + DNR
A4	M	33	6.56	493.5	1.49	0.9575	ATRA + ATO + IDA
A5	M	76	57.44	829.5	0.57	0.3519	ATRA + DNR
A6	F	33	7.50	537	1.39	NA	ATRA + DNR
A7	M	64	1.50	169	1.85	1.7688	ATRA + ATO + IDA
A8	F	44	2.30	416.5	1.88	1.3570	ATRA + ATO + IDA
A9	F	34	3.80	434	1.72	NA	ATRA + ATO + IDA
A10	F	63	6.30	501.5	0.97	1.1272	ATRA + ATO + IDA
A11	M	28	64.60	1048	0.36	0.2464	ATRA
A12	F	24	1.05	225.5	1.95	NA	ATRA + ATO + IDA
A13	F	24	1.60	143	1.8	2.0794	ATRA + ATO + IDA
A14	M	30	7.60	221	1.61	0.8966	ATRA + ATO + IDA
A15	F	38	34.10	617	0.49	NA	ATRA + DNR
A16	M	21	2.30	378	1.15	NA	ATRA + ATO + DNR
A17	F	23	0.70	183	2	1.5293	ATRA + DNR
A18	M	48	2.60	331	1.27	NA	ATRA + DNR
A19	F	24	3.90	411	1.86	NA	ATRA + ATO + IDA
A20	F	34	9.70	612	1.31	NA	ATRA + ATO + DNR
A21	F	60	1.30	173	1.87	1.9294	ATRA + ATO + IDA
A22	F	50	1.60	199	1.91	NA	ATRA + ATO + IDA
A23	M	30	1.60	189	1.85	NA	ATRA + ATO + IDA
A24	F	56	1.10	191	1.99	NA	ATRA + ATO + IDA
A25	M	51	3.20	367	1.7	NA	ATRA + ATO + IDA
A26	F	24	2.20	336	1.91	NA	ATRA + ATO + IDA
A27	M	40	1.00	156	1.97	NA	ATRA + ATO + IDA
A28	M	15	28.30	729	0.93	NA	ATRA + ATO + IDA
A29	F	62	0.60	201	2.13	NA	ATRA + ATO + DNR
A30	M	50	41.40	854	0.64	NA	ATRA + ATO + IDA
A31	F	44	67.20	1137	0.3	0.3373	ATRA + ATO + IDA
A32	M	52	1.50	179	1.82	NA	ATRA + ATO + IDA

M: male; F: female; WBC: white blood cell; ATRA: *all  trans* retinoic acid; ATO: arsenic trioxide; DNR: daunorubicin; IDA: idarubicin; NA: not available.
